# Mechanotransduction and Metabolism in Cardiomyocyte Microdomains

**DOI:** 10.1155/2016/4081638

**Published:** 2016-12-04

**Authors:** Francesco S. Pasqualini, Alexander P. Nesmith, Renita E. Horton, Sean P. Sheehy, Kevin Kit Parker

**Affiliations:** ^1^Disease Biophysics Group, Wyss Institute for Biologically Inspired Engineering, School of Engineering and Applied Sciences, Harvard Stem Cell Institute, Harvard University, Cambridge, MA, USA; ^2^Institute for Regenerative Medicine (IREM), Wyss Translational Center, University and ETH Zurich, Zurich, Switzerland; ^3^James Worth Bagley College of Engineering and College of Agriculture and Life Sciences, Mississippi State University, Starkville, MS, USA

## Abstract

Efficient contractions of the left ventricle are ensured by the continuous transfer of adenosine triphosphate (ATP) from energy production sites, the mitochondria, to energy utilization sites, such as ionic pumps and the force-generating sarcomeres. To minimize the impact of intracellular ATP trafficking, sarcomeres and mitochondria are closely packed together and in proximity with other ultrastructures involved in excitation-contraction coupling, such as t-tubules and sarcoplasmic reticulum junctions. This complex microdomain has been referred to as the intracellular energetic unit. Here, we review the literature in support of the notion that cardiac homeostasis and disease are emergent properties of the hierarchical organization of these units. Specifically, we will focus on pathological alterations of this microdomain that result in cardiac diseases through energy imbalance and posttranslational modifications of the cytoskeletal proteins involved in mechanosensing and transduction.

## 1. Introduction

The dynamic range and durability of the heart are enabled by the hierarchical organization of sarcomeres, the organ's force-generating units [1]. Sarcomeres are highly ordered arrays of molecular motors developed and maintained through a finely regulated mechanotransductive mechanism, sarcomerogenesis [2]. A second mechanism, myofibrillogenesis, ensures that sarcomeres serially register along acting bundles, creating the parallel arrays of myofibrils responsible for the cardiomyocyte striated appearance [3]. Through dedicated cell-cell junctions, myofibrils register across multiple cardiomyocytes creating myocardial sheets that wrap themselves around the cardiac chambers ensuring efficient pumping of blood in the circulation [4, 5].

While the anisotropic organization of the contractile cytoskeleton ensures efficient organ-level contraction, large quantities of Ca^2+^ ions and adenosine-triphosphate (ATP) molecules are required at the subcellular level for sarcomere contraction. In fact, cardiomyocytes feature a network of dedicated calcium storages, known as sarcoplasmic reticulum, as well as a large number of mitochondria, the organelles responsible for synthesizing ATP molecules from a range of available energy substrates [6]. Specifically, while glycolytic mechanisms are sufficient to meet the organ's ATP requirements during development, an increased energy demand coupled with an abundance of energy dense fatty acids promotes a shift towards oxidative metabolism as the organism matures. Fatty acid oxidation requires a complex set of enzymes that cluster into mitochondria to effectively participate in the tricarboxylic acid cycle [7, 8].

To ensure rapid and efficient transfer of ATP molecules, mitochondria in cardiomyocytes localize in close proximity with sarcomeres, the sarcolemmal invaginations known as t-tubules, and the sarcoplasmic reticulum. This creates a functional microdomain, termed the intracellular energetic unit (ICEU), where rapid catabolism drives a chemical gradient of ATP from the mitochondria to the sarcoplasmic reticulum and sarcomeres [9, 10]. Here, we first review some recent results suggesting a link between the cell microenvironment, the contractile cytoskeleton, and metabolism, which we hypothesize to interact at the level of intracellular energetic units. We will then argue that pathological alterations of this microdomain, and not only of its components, result in cardiac diseases through both energy imbalance and the direct impairment of structural and contractile cytoskeleton components. Finally, we speculate that hiPS-derived cardiomyocytes and heart-on-a-chip platforms may be used to investigate the pathophysiology of cardiomyocyte intracellular energetic units.

## 2. Lessons Learned from Recent In Vitro Experiments

**Figure 1:**
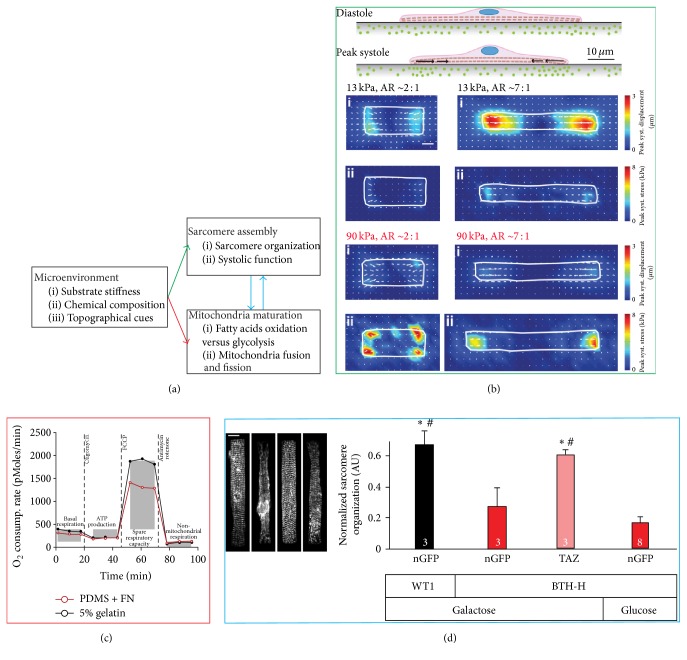
Mechanosensitive control of cardiomyocytes metabolism. (a) Putative link between microenvironmental cues, sarcomere assembly, and mitochondria maturation. (b) Traction force microscopy experiments suggesting that in response to stiffening of their microenvironment cardiomyocytes may regulate their shape to maintain an optimal work output. (c) Metabolic flux analysis showing the cardiomyocytes cultured on soft substrates stiffness (5% gelatin, black) retained a greater respiratory capacity than on stiffer ones (polydimethylsiloxane, PDMS, red). (d) Qualitative and quantitative analysis of cardiomyocytes structure in a disease-on-a-chip study of Barth syndrome, an inherited cardiomyopathy caused by a mutation to the tafazzin gene. The analysis showed that hiPS-derived cardiomyocytes obtained from a Barth syndrome patient (BTH-H, nGFP) had significantly impaired contractile architecture with respect to cardiomyocytes obtained from a healthy individual (WT1, nGFP). Furthermore, introducing wild-type tafazzin (BTH-H, TAZ), but not restoring the basal ATP level by switching to the glycolytic pathway (BTH-H, Glucose), in diseased cells rescued the structural phenotype. Scale bar 10 *μ*m. Together, these findings support the notion that mechanotransductive processes like sarcomerogenesis and myofibrillogenesis are linked not only to metabolism but also to mitochondria structure and function. Images were obtained with permission from the following sources: (b) McCain et al., 2014a [17]; (c) McCain et al., 2014b [20]; and (d) Wang et al., 2014 [21]. ^*∗*,#^Significant (*p* < 0.05) differences with respect to the nGFP-galactose and nGFP-glucose groups, respectively.

*“How do cardiomyocytes build themselves?”* [11] Following up on initial in vivo studies [12], in vitro and in silico assays were developed to characterize how the physicochemical characteristics of the cell microenvironment influence sarcomerogenesis and myofibrillogenesis [13–16]. In engineered isolated cardiomyocytes [17], cardiac microtissues [18], and laminar monolayers [19], microenvironmental cues constitute a chemomechanical signal that controls cell shape and the registration of the cell contractile apparatus. Intriguingly, recent results suggest that mechanotransduction may also affect cell metabolism [17, 20, 21] (Figure 1(a)) as we will describe in this section.

Hypertrophied and failing hearts have a higher fibronectin content and are less compliant than healthy organs, and their cardiomyocytes exhibit distinct morphological characteristics. In these pathological conditions, cardiomyocytes change not only in size, but also in aspect ratio—their length-to-width ratio changes from ~7 : 1 in healthy hearts to ~3 : 1 and ~11 : 1 in concentric and eccentric hypertrophy, respectively [22–24]. We hypothesized that cardiomyocytes in a diseased microenvironment remodel to preserve contractile function at the cellular level. To test this hypothesis, we developed a dedicated in vitro cell culture system. We engineered shape-controlled neonate rat cardiomyocytes on deformable gels of tunable stiffness. We then employed immunocytochemistry techniques, traction force microscopy, and a computational model of muscle mechanics to characterize the structure and function of the contractile apparatus and correlated them to the engineered cell shapes. We established that cardiomyocytes with normal length-to-width ratio exerted maximal contractile force on substrates with physiological, but not pathological, stiffness. On these diseased substrates, myocytes with a reduced length-to-width ratio (Figure 1(b)) exhibited better contractile performances. These results support the notion that cardiomyocytes remodel their shape, and therefore the relative amount of sarcomeres aligned in series or parallel to preserve their mechanical function in the presence of increasingly stiffer cell microenvironments [17]. In a separate study, we hypothesized that the fetal phenotype of stem cell derived cardiomyocytes could be improved by culturing these cells for an extended period of time in the presence of an adult-like engineered microenvironment [14, 25, 26]. We reasoned that heart development occurs over ~270 days in humans and therefore we hypothesized that long-term in vitro culture may coerce human stem cell derived cardiomyocytes towards a more mature phenotype. Unfortunately, traditional culture conditions utilize rigid materials from which stem cell derived cardiomyocytes tend to delaminate as they mature. We developed micromolded gelatin hydrogels to engineer rat primary and hiPS-derived cardiomyocytes into tissues that exhibit laminar architecture and mature and aligned sarcomeric structure and are functionally proficient. While the structural organization and functional proficiency of engineered neonatal rodent cardiomyocytes cultured on compliant gelatin hydrogels or stiffer fibronectin-coated polydimethylsiloxane (PDMS) substrates were similar [26, 27], we showed that myocytes had a greater spare respiratory capacity on soft than on stiff substrates (Figure 1(c)). These findings support the notion that microenvironmental cues, such as substrate stiffness and ECM coating, may impact cardiomyocyte metabolism independently from changes in cell structure or function [20].

Finally, we devised a disease-on-a-chip platform to study Barth syndrome using patient-specific hiPS-derived cardiomyocytes [21]. This disease is characterized by mutations of the gene encoding tafazzin, a protein involved in the maintenance of the mitochondrial inner membrane [28]. Patients develop a metabolic cardiomyopathy, but detailed molecular mechanisms and possible therapeutic strategies remain unknown. We hypothesized that impaired mitochondrial structure would cause mitochondrial dysfunction leading to disrupted sarcomeric structure and contractile function. To test this hypothesis, we cultured patient-specific hiPS-derived cardiomyocytes on our heart-on-a-chip platform, to support establishment of laminar tissue architecture and to enable direct measurement of contractile stress generation. Metabolic dysfunction was exacerbated by culturing tissues in a media containing galactose to prevent compensation via glycolytic pathways. In agreement with our hypothesis, engineered Barth cardiac tissues were characterized by depressed respiratory capacity, unorganized sarcomere structure, and decreased contractile twitch and peak systolic stress. Reintroduction of wild-type transcripts [29] for tafazzin was sufficient to rescue the disease phenotype structurally and functionally, confirming the causative role of mitochondrial dysfunction in this myopathy. Interestingly, when galactose was replaced with glucose, the basal whole-cell ATP level in diseased and healthy cardiomyocytes was rescued, but the structural impairment of the contractile cytoskeleton persisted, indicating that the ATP deficit was not the etiology of cardiac dysfunction in Barth syndrome (Figure 1(d)). Importantly, when we treated disease cells with ROS-scavengers we were able to rescue the disease phenotype, suggesting that the ROS buffering system in cardiomyocytes may be an overlooked target for pharmacological interventions in Barth syndrome. These results support the notion that mitochondria dynamics affect myofibrillogenesis not only by meeting the energy demand, but also through ROS-mediated mechanisms [21].

Taken together, these findings point to mechanosensitive feedback loop through which the cell microenvironment modulates the cross-talk between cardiomyocyte cytoskeletal architecture and metabolic profile, ultimately affecting the heart's ability to function (Figure 1(a)).

## 3. Intracellular Energetic Units in Healthy Hearts

**Figure 2:**
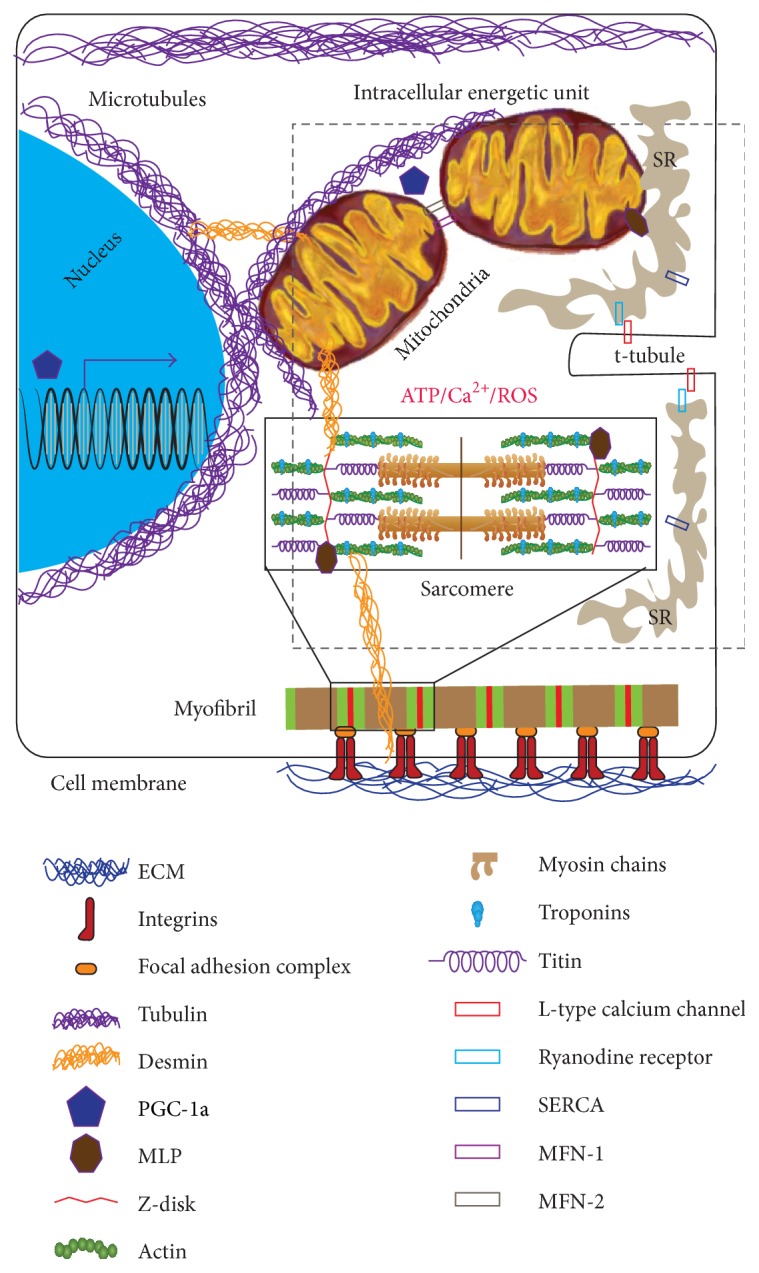
Schematic representation of the intracellular energetic unit. Mitochondria, sarcomeres t-tubules, and sarcoplasmic reticulum (SR) are closely packed together in a functional microdomain known as the intracellular energetic unit. Establishment and maintenance of these microdomains require tethering to a variety of cytoskeletal proteins that have been shown to participate in mechanotransduction. Furthermore, organelles and ultrastructures in the unit exchange through ATP, calcium ions, and reactive oxygen and nitrogen species.

We believe that the link between microenvironment, metabolism, and sarcomerogenesis resides in the mechanochemical signaling that occurs at the level of the intracellular energetic units, whose structural integrity is ensured by a variety of cytoskeletal elements previously implicated in mechanotransduction. Here we review the development of sarcomeres and mitochondria, as well as the establishment and maintenance of intracellular energetic units in cardiomyocytes (Figure 2).

### 3.1. Sarcomeres

In striated muscle cells, including cardiomyocytes, sarcomeres are serially arranged along parallel arrays of myofibrils. Their orchestrated activation is responsible for cell contraction and their physicochemical state determines how the organ contractile force changes in response to environmental challenges, such as pre/postload [30–32]. The correct spatial localization of sarcomeres, as well as their continuous renewal and remodeling along striated fibers, is ensured through the processes of sarcomerogenesis and myofibrillogenesis [33–35]. These processes start with the recruitment of integrins in focal adhesions near the cell membrane; these adhesions anchor the cell cytoskeleton to the extracellular matrix allowing actin filaments to extend outwards [22]. As the focal adhesions mature through the recruitment of additional integrins and signaling kinases, premyofibrils rich in Z-bodies (immature assemblies of contractile proteins) form near the spreading cell edge [36, 37]. Myosin, actin, tropomyosin, and various troponin isoforms polymerize and interweave to form sarcomeres via template proteins, nebulin and titin [37]. Thick and thin filaments are subsequently assembled [38] and terminated by actin capping proteins [12, 35, 37]. As the myofibrils mature and increase in width, linear arrays of Z-bodies fuse to form Z-bands [36]. These are further anchored by costameres and attachment sites that transmit force between the cell cytoskeleton and the extracellular matrix [39], thus closing the mechanotransductive feedback loop.

### 3.2. Mitochondria

Approximatively 35% of the volume of an adult cardiomyocyte is occupied by mitochondria, interspersed within the cell contractile apparatus [6]. To achieve the number and spatial distribution observed in adult cells and to limit the degradation of their DNA, mitochondria continuously merge together (fusion) and split apart (fission) in a process termed mitochondrial biogenesis [40]. Chief among the molecular mediators of this process is the coactivator PGC-1*α* (peroxisome proliferator-activated receptor *γ*—PPAR*γ*—coactivator 1*α*) [41]. Downstream of PGC-1*α*, a family of large GTPases known as dynamin-related proteins (DRPs), further controls the fission and fusion cycles [42]. Mitochondrial fission requires helical DRPs known as Dnm1/Drp1 that wrap themselves around the mitochondrial membrane and, by hydrolyzing GTP, constrict the membranes together until they divide [43–45]. Mitochondrial fusion occurs in two phases: membrane tethering and lipid mixing [46]. These events are regulated by additional DRP such as mitofusins (Mfn-1 and Mfn-2) and OPA1/Mgm-1 [47–49].

During development, biogenesis affects structure, function, and tethering of mitochondria as it requires the remodeling of the organelle inner and outer membranes, as well as of the receptors and enzymes located in the cristae [50]. In immature cardiomyocytes, mitochondria begin as fragmented bodies with poorly developed cristae and are located primarily in the perinuclear regions of the cytoplasm of immature cells [51, 52]. As cardiomyocytes mature, mitochondria transform into organized tubular structures containing elongated cristae and display elevated mitochondrial membrane potential, increased oxygen consumption, and elevated ATP production relative to the fragmented structures observed in immature cells [51, 53]. Importantly, a study using embryonic mouse hearts demonstrated that the mitochondrial permeability pores are open during the early stages of heart development, and their closure correlates with cytoskeletal maturation [54]. Furthermore, the authors demonstrated that pore closure facilitated myofibrillogenesis completion by reducing the cytoplasmic levels of reactive oxygen species.

### 3.3. The Intracellular Energetic Unit

The interplay between sarcomeres and mitochondria is critical to myocardial function. During cell contraction, calcium ions and ATP molecules are consumed to ensure cross-bridge cycling, muscle shortening, and the development of contractile force [38, 55]. Efficient transfer from energy production sites to energy consumption sites has been evolutionarily achieved by creating a functional microdomain, the intracellular energetic unit [56–58]. That is, mitochondria, sarcomeres, t-tubules, and the sarcoplasmic reticulum are packed so close together that, during normal contraction, calcium ions and ATP molecules are released and uptaken in the intracellular energetic unit before they have the time to diffuse outside of it. There are at least two ways for this arrangement to impact cardiomyocyte mechanotransduction. First, mitochondrial function is necessary to ensure the energy demands of myofibrillogenesis and sarcomerogenesis are met [59, 60]. Secondly, tethering of the mitochondrial membrane to the cell cytoskeleton is required to facilitate nucleotide channeling [58] and the movement of metabolites involved in oxidative phosphorylation [57, 61]. For example, microtubules [62] and actin [63] have been implicated in long- and short-distance mitochondrial trafficking, respectively. Further, tethering to tubulin, actin [64], and intermediate filaments [65] is required for mitochondrion cristae to acquire their characteristics invaginated morphology. Tubulin tethering to mitochondria has been shown to play both structural and functional roles in striated muscle homeostasis [66] and disease [10]. Specifically, by physically and chemically interacting with the mitochondrial outer membrane, tubulin modulates the function of the permeability transition pore [67] and of voltage-dependent anion channels [68]. Mitochondrial tethering to cytoskeletal proteins involved in mechanosensing represents a mechanism through which the extracellular matrix may affect the contractile and metabolic apparatuses. In fact, integrins connect the cell cytoskeleton to the extracellular matrix at specific locations known as focal adhesions [22]. External mechanical forces activate the integrin-mediated response of protein-tyrosine kinases of the Src family and cytoplasmic form of focal adhesion kinases (FAK) and mitogen-activated protein kinases (MAPK) [69, 70]. Importantly, integrin signaling mediated by Rho family GTP-binding proteins was shown to directly affect mitochondria structure and function [71].

Finally, recent studies suggest that sarcomeres and mitochondria directly exchange internal forces through sarcomeric shortening and mitochondrial swelling [72, 73]. In fact, mitochondria located in between sarcomeres are squeezed during cell contraction, thus providing an intracellular load against which force must be generated [73]. At the same time, stimuli that lead to mitochondrial swelling cause these organelles to apply pressure against neighboring structures [72]. For example, through the microtubule network, swollen mitochondria severely deformed the nuclear shape, possibly initiating chromatin remodeling as well as epigenetic and transcriptional events [74].

Taken together these results suggest that the intracellular energetic unit itself can sense and respond to changes in the cell microenvironment through its cytoskeleton components.

## 4. Intracellular Energetic Units in Diseased Hearts

**Figure 3:**
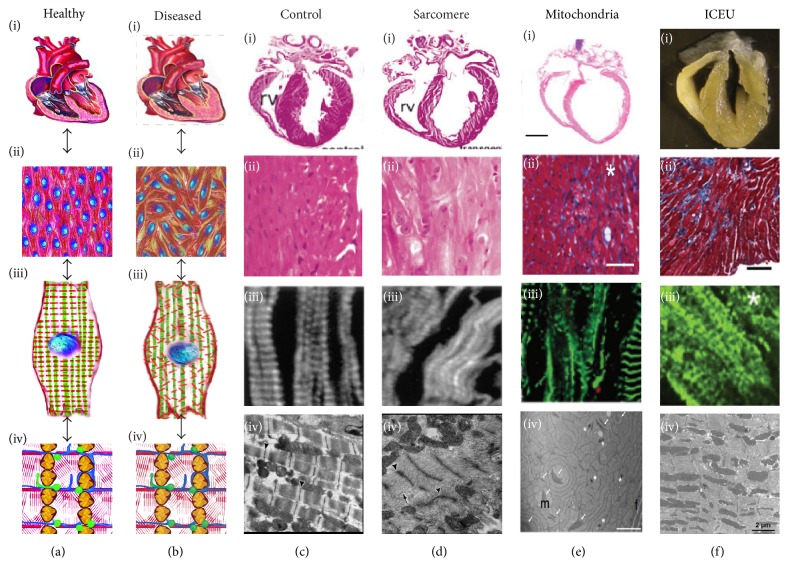
Multiscale disease mechanism in the heart. (a-b) Schematic representation of the multiscale hierarchical organization of sarcomeres in the healthy (a) and diseased (b) heart: the structure of the ventricular wall (i) is affected by the alignment of the myocytes within the myocardial layers (ii) that is modulated by the orientation of myofibrils within cardiomyocytes (iii), which in turn depends on the organization of the intracellular energetic units (iv, ICEUs). (c–f) Examples of organ (i), tissue (ii), cell (iii), and ICEU (iv) level structure in healthy control animals (c), as well as diseased animals with dysfunctional sarcomeres (d), mitochondria (e), and intracellular energetic units (f). Images were obtained with permission from the following sources: (c, d) Sussman et al., JCI, 1998 [85]; (e-i, ii) Krebs et al., PNAS, 2011 [126]; (e-iii) Zaglia et al., JCI, 2014 [93]; (e-iv) Perdomini et al., Nat Med, 2014 [127]; (f-i) Chen et al., Circ Res, 2012 [128]; (f-ii) Martin et al., Circ Res, 2014 [125]; (f-iii) Purevjav et al., JACC, 2010 [121]; and (f-iv) van den Bosch et al., Cardiovasc Res, 2005 [117]. Arrows and asterisks indicate pathologically disorganized sarcomeres, mitochondria, and ICEUs.

In the traditional view, the heart structure and function are ensured by the multiscale hierarchical organization of sarcomeres that are closely packed with mitochondria (Figures 3(a)-3(b)). Mutations to proteins involved in cell contraction and metabolism result in a variety of inherited cardiomyopathies, characterized by maladaptive remodeling at the organ, tissue, and cell level and by disorganized intracellular energetic units (Figures 3(c)–3(e)). If the intracellular energetic units are responsible for cardiac homeostasis, we reasoned that mechanotransductive alterations to their structure and function should result in similar disease phenotypes (Figure 3(f)). Here we first review animal and clinical studies relative to inherited diseases affecting the contractile and metabolic apparatuses, respectively. We then present a number of conditions that were linked to the dysfunctional interplay between sarcomeres, mitochondria, and the mechanosensitive constituents of the intracellular energetic unit. Finally, we discuss the possible pathological implications of excess reactive oxygen species in the intracellular energetic units.

### 4.1. Sarcomerogenesis in Diseased Hearts

Many inherited cardiomyopathies have been linked to genetic defects in sarcomeric proteins such as myosin heavy chain (MHC), myosin light chain (MLC), cardiac troponin T (cTnT), and cardiac troponin I (cTnI) [75, 76]. Disease pathways are complex in that different mutations of the same sarcomeric protein can give rise to both hypertrophic and dilated cardiomyopathies [76]. In fact, abnormalities in sarcomeric proteins such as actin, titin, myosin and myosin-binding proteins, and cTnT impaired sarcomerogenesis and led to severe cardiac impairment, abnormal chamber morphology, and congenital heart disease [77–81].

Heart pathologies are often characterized by sarcomere disarray which leads to compromised contractile function. Titin is a major sarcomeric protein that acts as a template for myosin incorporation during sarcomerogenesis. The absence or mutation of the gene encoding for titin resulted in disruptions in sarcomerogenesis and dysfunctional sarcomeres which ultimately led to heart failure [82]. Myosin light chain kinases (MLCK) regulate sarcomerogenesis through the phosphorylation of ventricular MLCK2. Patients with dilated cardiomyopathy exhibited reduced ventricular MLCK2 expression, unstable myofilaments, and defective myosin thick filaments assembly [83]. Additionally, reduced cardiac MLCK levels in zebrafish embryos result in dilated ventricles with thinned walls and poor sarcomere assembly [84]. Finally, tropomodulin—a tropomyosin-binding protein—regulates actin thin film length and is essential for sarcomere maintenance [85]. Alterations in tropomodulin expression levels disrupt sarcomere structure [86], whereas overexpression can lead to the development of juvenile dilated cardiomyopathy due to myofibril disassembly [85].

Besides their function in contraction, specific myofibrillar and sarcomeric associated proteins act as sensors and transducers of internal strains. For example, cardiac troponin mutations disrupt sarcomere assembly [87] and calcium regulation resulting in arrhythmias, sudden death, and cardiomyopathies [88, 89]. Cardiac troponins' loss-of-function alters the regulatory role of the troponin complex leading to an increase in calcium sensitivity in hypertrophic cardiomyopathy and a decrease in sensitivity in dilated cardiomyopathy [90, 91]. Mutations in cTnI have also been linked to restrictive cardiomyopathy and result in greater calcium sensitivity of cardiac myofilaments [92].

Taken together, these results implicate sarcomere integrity and structural organization in heart function, as compromised sarcomere structure triggers organ-level disease development.

### 4.2. Bioenergetics and Biogenesis in Diseased Hearts

Heart function depends heavily on ATP generation; therefore impediments or alterations in metabolism inevitably lead to cardiomyocytes apoptosis through autophagy and contractile dysfunction [93–95]. The metabolic state of the heart can be gauged estimating the extent to which the organ relies on oxidative (mature) and glycolytic (fetal) pathways [96–98]. Alterations in energy substrate utilization and energy metabolism, including mitochondrial dysfunction and increased glucose utilization, are characteristic of the hypertrophied and failing myocardium [8, 99]. Following stress, that is, chronic pressure, volume overload, or infarction, the heart gradually loses the ability to generate a sufficient supply of ATP, prompting organ failure [100, 101]. In the early stages of heart failure, fatty acids are the predominant myocardial energy substrate. However, as the disease progresses, there are a concomitant increase in glycolysis and glucose oxidation, a decrease in fatty acid oxidation, and a reduction of respiratory chain activity which resembles the fetal metabolic profile [98, 102, 103]. Under pressure overload hypertrophy, the fetal metabolic profile is reactivated with an increase in glycolysis and a decrease in fatty acid oxidation in response to increased workload [104, 105]. Interestingly, the notion that these pathological changes were* exclusively* associated with lack of ATP was challenged by a study on mice completely lacking creatine—and therefore phosphocreatine—a key indicator of energy depletion in the heart [106]. While the role of creatine kinase pathways remains essential for myocardial homeostasis and disease [107], the creatine-deficient animals responded reasonably well to myocardial infarction and pressure overload suggesting that energy depletion may not be the dominant mechanism in heart failure. Instead, and in agreement with our own observations in vitro, accumulation of ROS [108] and other intermediate metabolites [109] may be responsible for pathological responses to hemodynamic overload and the onset of heart failure.

Abnormalities in cardiac energy metabolism have also been associated with the loss of mitochondrial structural integrity [110, 111], maintained through mitochondrial biogenesis [112]. For example, knocking out Mfn1 and Mfn2 in mice caused lethal heart failure, as it abolished mitochondrial tethering and fusion of the outer membrane, leading to fragmented, smaller, and poorly organized mitochondria [113]. Furthermore, mitochondria exhibited reduced size and a decline in structural integrity in the failing human myocardium [105]. Similarly, round mitochondria that lacked organization within the cytoskeleton were observed in dilated cardiomyopathy and hibernating myocardium [80, 114]. Pigs with hypertrophic cardiomyopathy were shown to have swollen cardiac mitochondria with disrupted cristae and disoriented myofibrils resulting from the internal forces exerted by mitochondrial growth [111]. Finally, mitochondria of failing hearts in rats are characterized by sparse and disorganized cristae [115]. These alterations in mitochondria morphology suggest interruptions in mitochondrial fusion and fission and suggest that mitochondria biogenesis is essential for structural organization.

Together, these studies suggest that maintenance of the correct mitochondrial structure and function is essential to cardiac homeostasis.

### 4.3. Intracellular Energetic Units in Diseased Hearts

Impairment of the structural interactions between components of the intracellular energetic unit also leads to cardiomyopathies. For example, muscle LIM protein (MLP), a mechanosensor in the Z-disc of myofilaments, has been shown to interact directly with metabolic enzymes [116]. In mice with MLP deficiency, mitochondria density around the myofilaments was reduced [117, 118]. Similarly, intracellular energetic unit disruption also led to impaired mitochondrial ATP channeling to the sarcoplasmic reticulum, due to the increased distance between mitochondria and the sarco/endoplasmic reticulum calcium-ATPase (SERCA) pumps [94, 118]. This suggests that cytoskeletal MLP may play a role in the sensing mechanisms that link mitochondria and the cell cytoskeleton [119, 120]. Similar studies with desmin-null mice hearts revealed that cytoskeletal disorganization led to looser packing of mitochondria within the myofibrils and sarcoplasmic reticulum [119] and impaired ATP transfer and myocardial dysfunction [10, 118]. Finally, cardiomyopathies caused by mutations in desmin and other intermediate filament related proteins are characterized by myofibril disruption [24, 94, 110, 121, 122].

Additionally, disruption of normal sarcomere function directly impairs metabolic function, an initial indication of disease. In a transgenic rat model overexpressing a mutated cTnT [123], rats exhibited normal contractile function and no hypertrophy compared to controls. After pretreatment with glucose-deprived media, transgenic rats showed decreased metabolic reserve resulting in reduced cardiac fractional shortening, contraction, and relaxation velocity. Similarly, in a group of patients diagnosed with familial hypertrophic cardiomyopathy due to mutations in *β*-myosin heavy chain (*β*-MHC), cTnT, or myosin-binding protein C (MyBPC) [124], altered cardiac metabolism was observed in both symptomatic and asymptomatic patients. The finding that altered cardiac metabolism occurred in patients with normal echocardiographs suggests that altered metabolism may be a cause, not an effect, of cardiac hypertrophy [125]. Additionally, disruption of normal mitochondrial function can also drive changes in cardiac structure and function, resulting in significant disease. For example, homozygous mutation to Med30zg, a mediator of gene expression, led to decreased transcription of genes requisite for oxidative phosphorylation and subsequently caused lethal cardiomyopathy in a mouse model [126]. Histological characterization of the myocardium indicated fibrosis, necrosis of cardiomyocytes, and dilation of both ventricles. Further, ultrastructural analysis showed loss of Z-disk organization demonstrating a critical role for metabolic regulation of cardiac structure over a range of spatial magnitudes. In a mouse model of Friedreich's ataxia [127], a mutation of the mitochondrial protein frataxin involved in oxidative phosphorylation resulted in the progressive development of hypertrophic cardiomyopathy.

Even when the structural integrity of the ICEU and its components are preserved, changes in calcium utilization patterns may lead to poorly functional microdomains. In fact, the sarcoplasmic reticulum is physically tethered to mitochondria through a variety of mechanisms [9], including Mfn-1 and Mfn-2 [128]. And this tethering may play a role in disease onset and progression; for example, Mfn-1/2 are downregulated in heart failure impairing mitochondrial fusion and SR function, which in turn lead to decreased SR calcium load and, over time, to decreased calcium release. Mitochondrial calcium uptake will also be affected at this point, as regeneration of pyridine nucleotides is impaired resulting in reduced antioxidative capacity [129] and increased oxidative stress-mediated myocardial remodeling [108, 130].

As a consequence of the critical coupling between sarcomere and mitochondria, new therapeutic strategies have emerged that target various aspects of metabolism in order to restore the mechanical function of the heart [131]. In a study of patients with nonobstructive hypertrophic cardiomyopathy [132], pharmacological treatment with perhexiline decreased fatty acid oxidation and increased glucose utilization as an energy substrate [131]. Patients experienced improved exercise capacity and overall decreased symptoms compared to placebo controls, suggesting that changes in metabolic substrate utilization may play an important role in disease progression. At the same time a number of clinical trials aimed at repurposing drugs that were approved for the treatment of metabolic disorders in other organs were not successful [133]. For example, Acadesine [134], a nonspecific agent targeting AMP-dependent protein kinase, L-arginine [135], an alternative energy substrate utilized in the treatment of other mitochondrial myopathies, and adenosine receptor agonists [136, 137] did not improve the conditions of myocardial infarction patients.

Together these studies demonstrate that impairment of the interaction between contractile and metabolic components in the intracellular energetic unit triggers disease development through both ATP dependent and ATP-independent mechanisms.

### 4.4. Evidence for a ROS-Mediated Disease Mechanism

In addition to ATP exchange, sarcomeres and mitochondria interact through calcium ions and reactive oxygen and nitrogen species (ROS for convenience). A number of reviews are available describing how mitochondria sequester calcium ions from the cytosol, directly competing with cardiac contractility, calcium handling, and biochemical signaling [8, 138–140]. Similarly, ROS are a byproduct of mitochondrial metabolism [141, 142] that can alter cardiac function [143, 144] and activate the cell apoptotic program [95]. Additionally, impairment of the ROS buffering system causes oxidative damage to ionic channels in the plasma membrane [145], sarcoplasmic reticulum [143, 146, 147], and alterations in signaling cascades involving calcium/calmodulin-dependent protein kinase II (CaMKII) [148], protein kinase-C [149], Rho-associated protein kinase (ROCK) [150] and nicotinamide adenine dinucleotide phosphate (NADPH) oxidase 2 (NOX2) [151]. Importantly, recent studies further point towards a direct, beat-to-beat dependence of mechanotransduction, metabolism, and contractility: as myocyte stretching [152] and increased afterload [153] induced NOX and NOS dependent ROS signaling that directly affected calcium release from the SR and contractility. Interestingly, computational models predict that these effects may be proarrhythmic causing action potential shortening [154] and altering automaticity [155]. Here we focus on posttranslational modifications of cytoskeletal proteins that can impact the formation and maintenance of competent intracellular energetic units [142, 156].

ROS damage to cytoskeletal proteins involved in cardiomyocytes contractility directly impacts cell and organ function [157]. For example, elevation of ROS depresses contractile function due to (i) reduced tropomodulin-actin binding in the thin filament and (ii) increased F-actin depolymerization leading to reduced cross-bridge sliding velocity [158, 159]. Furthermore, *α*-actinin polymerization kinetics is also depressed in the presence of elevated ROS levels, which destabilized the Z-disks and compromised longitudinal force transmission [156]. Moreover, excess ROS enhance the activity of matrix metalloproteinase-2 (MMP-2), which has been directly linked to increased degradation of myosin light chain isoforms [160, 161].

Additionally, damage to the proteins that maintain the structural integrity of the intracellular energetic unit may have pathological relevance. For example, titin oxidation decreases the extensibility of the N2B domain [162], which has been linked to diastolic dysfunction [163]. Similarly, ROS-mediated damage to the mitochondria-microtubules connection has been observed after ischemic damage [164] and in volume overload [165]. A tethering of mitochondria to the SR has been postulated [166], and ROS-mediated damage to the microtubule network has been associated with calcium sparks abrogation [167] and decreased velocity of calcium wave propagation [168].

Taken together, these results suggest that the inability of cardiomyocytes to buffer excessive ROS production may result in extensive damage to the architecture and therefore to the functional proficiency of the intracellular energetic units. This is consistent with our Barth syndrome-on-a-chip study, in which we observed that treating the cells with a ROS scavenger resulted in complete rescue of the structural and functional disease phenotype [21]. Furthermore, these findings are consistent with the observation that closure of the mitochondrial transition pore facilitates maturations of the contractile cytoskeleton in embryonic cardiomyocytes by lowering the cytoplasmic concentration of ROS [54].

## 5. Human Diseases-on-Chips and the Intracellular Energetic Units

If intracellular energetic units control cardiac homeostasis and disease,* “How do these microdomains build themselves? How do they adapt to changes in the cell microenvironment?”* Unfortunately, these questions are difficult to answer utilizing traditional animal models for a number of reasons: (i) humans and animals preferentially express distinct isoforms of proteins involved in the structure and function of intracellular energy units, such as *α*- and *β*-myosin heavy chains; (ii) the interaction between organelles and ultrastructural units within functional microdomains occur at spatial scales that are difficult to study with animal model; and (iii) altering the microenvironment of cardiomyocytes in vivo causes a systemic organ-level response that confounds the analysis. Here we review how patient-specific human induced pluripotent stem cells (hiPSCs) and hiPSC-derived cardiomyocytes may be utilized to study the molecular mechanisms responsible for the development and maintenance of intracellular energetic unit in humans. Moreover, engineered tissue-culture platforms, so called hearts-on-chips, can be adopted to recapitulate healthy and diseased microenvironmental conditions in vitro and measure how these variables affect structure and function of human induced pluripotent stem cell derived cardiomyocytes.

### 5.1. Human Induced Pluripotent Stem Cell Derived Cardiomyocytes

Technologies for reprograming somatic cells into pluripotent stem cells [169] and directing their differentiation towards the cardiomyogenic phenotype [170] enabled the creation of in vitro replicas of various human diseases including channelopathies, vasculopathies, and cardiomyopathies [171, 172]. For example, hiPS-derived cardiomyocytes carrying mutations to troponin T (TNTT2) and *β*-myosin heavy chain (MyH7) recapitulated in vitro dilated and hypertrophic cardiomyopathy, respectively [173, 174]. Interestingly, the intracellular energetic units were severely underdeveloped in these hiPSC-derived cardiomyocytes that displayed reduced myofibrillogenesis and immature, sparse mitochondria. Moreover, hiPSC-derived cardiomyocyte carrying mutations to plakoglobin [175] and plakophilin-2 [176–178] recapitulated in vitro arrhythmogenic right-ventricular dysplasia/cardiomyopathy (ARVD/C). This is a rare inherited disease characterized by pathological fat infiltration, fibrosis, and cardiomyocyte loss predominantly in the right ventricle resulting in right-ventricular dysfunction and ventricular arrhythmias [179]. The mutated plakophilin-2 coactivated both normal PPAR*α*-dependent metabolism and abnormal PPAR*γ* pathway ultimately resulting in exaggerated lipogenesis, apoptosis, and impaired calcium handling. Furthermore, a different set of mutations to plakophilin-2 were associated with a Brugada syndrome phenotype both in patients and in patient-specific hiPSC-derived cardiomyocytes [180]. Interestingly, at the ultrastructural level, these symptoms were associated with alterations to various components of the intracellular energetic unit, including intercalated disk and microtubules. In addition, hiPSC-derived cardiomyocytes carrying mutations to ryanodine receptors (RyR2) calcium/calmodulin-dependent protein kinase II (CaMKII) and calsequestrin (CLSQ) displayed calcium oversensitization, the hallmark of catecholaminergic polymorphic ventricular tachycardia (CPVT) [181–183]. Specifically, abnormalities in whole-cell calcium transients were observed, including proarrhythmic early/after depolarization events [181]. Interestingly, more abundant and longer local spontaneous releases of calcium were observed at spatial scale comparable to the intracellular energetic units [128, 183]. Computational studies suggest that this extra calcium may have functional implications for sarcomere and mitochondrial function and thus implicate the dysregulation of the intracellular energetic units in disease development [184].

Furthermore, mutations to either chromosomal or mitochondrial genes involved in cardiomyocyte metabolism also result in cardiomyopathies. For example, hiPSC-derived cardiomyocytes have been obtained for patients affected by Friedreich ataxia, a neurodegenerative disorder associated with hypertrophic cardiomyopathy [185]. In this model, deregulation of iron homeostasis leads to disorganized mitochondrial network and mitochondrial DNA depletion, which in turn results in energy deficiency that blunt the calcium cycling and contractile properties of cardiomyocytes. Similarly, in a recently proposed in vitro model for Pompe disease, hiPSC-derived cardiomyocytes exhibited reduced metabolic capacity associated with aberrant mitochondria morphology [186]. Together these studies suggest that hiPSC-derived cardiomyocytes can also be utilized to model dysregulation of the ATP local homeostasis in the intracellular energetic units responsible for the onset and development of various cardiomyopathies.

We previously argued that elevated ROS levels impair the mechanosensitive processes responsible for the genesis and maintenance of the intracellular energetic units. A number of hiPSC-based disease models appear to support this notion. For example, hiPSC-derived cardiomyocytes recapitulating genetically or environmentally caused diabetic cardiomyopathy exhibited deranged intracellular energetic units, with immature sarcomerogenesis and impaired mitochondrial biogenesis [187]. In this study, similarly to what we observed with hiPSC-based model of Barth syndrome [21], ROS-generation, and not a metabolic insufficiency, triggered the disease. Moreover, hiPSC lines were derived from patients exhibiting ROS-mediated damage to proteins of the intracellular energetic unit. For example, hiPSC-derived neurons obtained from patients affected by Parkinson's [188] and Huntington's [189] diseases exhibited deregulation of the peroxisome proliferator-activated receptor c coactivator 1 (PGC-1*α*) and dynamin-related protein 1 (DRP1) pathways, involved in mitochondrial fusion and fission, respectively. Similarly, mutations to parkin [190] and plakophilin-2 [180] have been shown to destabilize the microtubule network in hiPSC-derived neurons and cardiomyocytes, respectively.

Taken together this body of evidence suggests that patient-specific hiPSC lines that carry mutations to proteins of the intracellular energetic units, as well as genome editing techniques to engineer isogenetic hiPSC lines carrying arbitrary mutations [191], are promising tools to study the regulation of intracellular energetic units in a human genetic background.

### 5.2. Cardiac Tissue Engineering and Heart-on-a-Chip Platforms

The pathophysiological state of the heart results from the interaction between the genetic instructions cardiomyocytes carry from birth and the constantly evolving microenvironment in which they operate. While access to hiPSC lines that carry a variety of genetic mutations is a fundamental step in designing in vitro models to understand disease mechanisms mediated by intracellular energetic units, the physicochemical characteristics of healthy and diseased cell microenvironments must also be captured in vitro.

While we have not engineered a complete biomimicry of the native architecture of the myocardium that incorporates the transmurally changing alignment of the myofibrils [192], a number of two- and three-dimensional in vitro analogs have been developed. These platforms can be distinguished for the degree of control they offer over the cell microenvironment. For example, 3D collagen sponges [193] and biowires [194] of variable stiffness were utilized to improve the maturation of primary and stem cell derived cardiomyocytes. Alternatively, cardiac patches coated with various extracellular matrix proteins have been shown to increase the maturation of pluripotent stem cell derived cardiomyocytes and to permit contractility studies via traditional force-transducers [195]. One extremely sophisticated extension of these efforts was recently published that incorporates an engineered vascular network and can be surgically implanted into the native myocardium [196]. Among nonvascularized 2D and thin-3D platforms, microstructured chambers coated with extracellular matrix proteins, and dog-bone shaped thin gels promoted the alignment of hiPSC-derived cardiomyocytes. In these studies, cells were genetically engineered to express a calcium-sensitive fluorescent protein, enabling the assessment of contractility and calcium cycling parameters in vitro [197, 198]. Additionally, micropillars-based platforms were manufactured in a range of pathophysiological stiffness to study single cardiomyocytes [199] and tissues [200]. Finally, cardiomyocytes were also cultured on densely packed arrays of microposts, coated with various extracellular matrix proteins [201, 202].

In our laboratory, we developed a heart-on-a-chip platform based on the muscular thin film technology [203]. In open-well or microfluidic chip, cantilevers were precut from a thin layer of synthetic material and microcontact printed [27] or micromolded [20, 204] to support a two-dimensional laminar sheet of cardiomyocytes. These hearts-on-chips were utilized to model Barth syndrome [21] and heart failure [19] as well as to assess the proficiency of stem cell derived cardiomyocytes [14, 26]. Furthermore, we optimized a traction force microscopy approach where cardiomyocytes are cultured on deformable hydrogels embedding fluorescent nanospheres that, similarly to the posts, can be tracked optically during cell contraction. Specifically, we employed a range of fabrication and surface-chemistry techniques to independently control substrate stiffness, extracellular matrix composition, and topography. We utilized this technique to suggest that remodeling of cardiomyocyte shape in hypertrophic cardiomyopathies may be a compensatory effect aimed at maintaining optimal contractile output [17]; that is, the establishment of cell-matrix adhesion at the cell-cell interface is a characteristic of immature, diseased, or regenerating cardiac tissues [18, 205].

Together, these efforts suggest that advanced platforms for cardiac cell and tissue phenotyping can be utilized to study intracellular energetic units. One challenge will be the integration of superresolution microscopy techniques [206, 207] that can resolve many different proteins and organelles [208] (actin [209], tubulin [210], and mitochondria [211]) in intracellular energetic units whose spatial extent is below the resolution limit of traditional microscopy.

## 6. Conclusions

In conclusion, we believe that the interactions between sarcomeres and mitochondria not only affect cardiomyocyte excitation-contraction coupling directly, but also participate in mechanotransduction, through the cytoskeletal proteins involved in the development and maintenance of intracellular energetic units. Alterations in the structure and function of these microdomains, such as those generated by oxidative posttranslational modifications, represent a disease mechanism that can now be better investigated integrating recent advances in stem cell biology and tissue engineering.
